# A mathematical model of multisite phosphorylation of tau protein

**DOI:** 10.1371/journal.pone.0192519

**Published:** 2018-02-06

**Authors:** Alexander Stepanov, Tatiana Karelina, Nikolai Markevich, Oleg Demin, Timothy Nicholas

**Affiliations:** 1 InSysBio, Moscow, Russia; 2 Pfizer Global R&D, Groton, Connecticut, United States of America; McGill University, CANADA

## Abstract

Abnormal tau metabolism followed by formation of tau deposits causes a number of neurodegenerative diseases called tauopathies including Alzheimer’s disease. Hyperphosphorylation of tau protein precedes tau aggregation and is a topic of interest for the development of pharmacological interventions to prevent pathology progression at early stages. The development of a mathematical model of multisite phosphorylation of tau would be helpful for searching for the targets of pharmacological interventions and candidates for biomarkers of pathology progression. In the present study, we for the first time developed a model of multisite phosphorylation of tau protein and elucidated the relative contribution of kinases to phosphorylation of distinct sites. The model describes phosphorylation of tau or PKA-prephosphorylated tau by GSK3β and CDK5 and dephosphorylation by PP2A, accurately reproducing the data for short-term kinetics of tau (de)phosphorylation. Our results suggest that kinase inhibition may more specifically prevent tau hyperphosphorylation, e.g., on PHF sites, which are key biomarkers of pathological changes in Alzheimer’s disease. The main features of our model are partial phosphorylation of tau residues and merging of random and sequential mechanisms of multisite phosphorylation within the framework of the probability-based approach assuming independent phosphorylation events.

## Introduction

Microtubule-associated protein tau (MAPT) stabilizes microtubules regulating cell architecture and cargo transport [1]. As an intrinsically disordered protein in its unbound state, tau adopts an ensemble of conformations more or less susceptible to different modifications. The most important events leading to pathological tau aggregation are the hyperphosphorylation, conformational changes, and truncation of native tau protein [2]. The sequence and hierarchy of the events are complex and cannot be traced in vitro and especially in vivo. At the same time, various events may facilitate pathological processes or protect from them. Hyperphosphorylation of tau may be regarded as an imbalance between kinases and phosphatases owing to either activation of tau kinases or inhibition of tau phosphatases. Multisite phosphorylation is an important mechanism for regulation of a protein’s function and life-time and triggers conformational changes that alter its interactions with other proteins. More than 80 residues of tau can be potentially phosphorylated by a number of kinases [3]. Hyperphosphorylation of tau is associated with tau aggregation into fibrils and neurofibrillary tangles correlating with the development of neurodegenerative diseases: various tauopathies including Alzheimer’s disease (AD). Indeed, in vitro experiments show that phosphorylation of tau protein accelerates its aggregation into fibrils [4] of which neurofibrillary tangles consist.

Glycogen synthase kinase 3 beta (GSK3β) is a proline-directed protein kinase generally considered one of the key players in tau phosphorylation. This kinase phosphorylates mainly (S/T)P motifs and specifically recognizes the (S/T)XXX(pS/pT) consensus motif whereby the first priming phosphorylation event is performed by GSK3β itself or another kinase. Priming (e.g., initial phosphorylation of S214 by the cAMP-dependent protein kinase [PKA]) may enhance or silence the catalytic efficacy of the kinase. Prephosphorylation by CDK5 increases subsequent phosphorylation catalyzed by GSK3β but not vice versa, i.e., no significant stimulation of phosphorylation is observed when phosphorylation by GSK3β is followed by phosphorylation by CDK5 [5]. As is the case for GSK3β, the activity of CDK5 is stimulated if tau is first prephosphorylated by PKA. Three residues (S396, S400, and S404) are phosphorylated by GSK3β sequentially: S404 first, then S400 and finally S396 [6]; this process can be regarded as GSK3β self-priming. Phosphorylation of GSK3β on Y216 increases its activity [7] and can occur as an autocatalytic process, which explains the good activity of recombinantly expressed GSK3β, but phosphorylation on S9 inhibits GSK3β activity [8]. Thus, priming means creating phosphorylation sites (inside recognized motifs) or enhancing the catalytic efficacy for further phosphorylation events because of conformational changes in the substrate.

Kinase p38γ, another candidate for pharmacological intervention, phosphorylates tau in vitro at four sites (S199, T205, S396 and S404) in a short-term reaction and hyperphosphorylates tau during long-term assays. T205 is a primary p38γ site in tau, and phosphorylation of T205 alleviates amyloid β toxicity in a mouse model of AD [9].

The tau phosphorylation state depends strongly on phosphatases. PP2A is the most abundant phosphatase in the human brain [10]. PP2A accounts for the majority of tau phosphatase activity among other brain phosphatases and is downregulated in AD. PP2A can also contribute to the tau hyperphosphorylation by influencing the kinase activity either directly or indirectly.

Pharmacological interventions in tauopathies and targeted tau hyperphosphorylation have been proposed. Several GSK3β inhibitors such as tideglusib, lithium, valproic acid, and AZD-1080 do not show improvements in cognitive outcomes in clinical studies [11]. A blocker of Fyn and related kinases (saracatinib) as well as VX-745, a kinase p38α-selective inhibitor, currently are in Phase II of clinical trials [12]. Inhibition of NUAK1, an AMPK-related kinase (exclusively phosphorylating tau at S356) decreases the total tau level and reverses the cognitive deficits in a mouse model of tauopathy [13].

**Fig 1:**
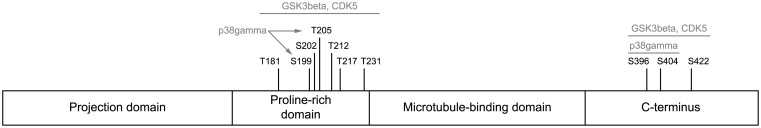
Schematic representation of the tau residues and kinases incorporated into the mathematical model.

Quantitative analysis of the contribution of kinases to the phosphorylation state of each residue should be helpful for searching for proper targets of pharmacological interventions and candidates for biomarkers of pathology progression or treatment efficacy. Ten tau sites and kinases incorporated into the model are presented in Fig 1.

Several mathematical models describing different outputs of a variety of molecular mechanisms involved in the processing of phosphorylation sites have been developed [14]. These approaches take into account the order and processivity of phosphorylation.

Compared to phosphorylation of a single residue, multisite phosphorylation increases the possibilities for regulating protein function considerably. A protein with *n* phosphorylable sites can exist in 2^n^ phosphorylation microstates, which may have different functional characteristics. Explicit mathematical description of such states by usual kinetic methods and differential equations is impossible due to combinatorial complexity. Various approaches have been developed to describe such complex systems, e.g., rule-based approaches [15]. These approaches have been applied to the analysis of signaling pathways with combinatorial complexity. Authors of these approaches announced that combinatorial explosion makes the traditional modeling paradigm (based on systems of differential equations) impractical [16, 17]. In contrast, agent-based or concurrent languages, such as kappa [16] or the closely related BioNetGen language [17] allow researchers to describe biological interactions in terms of some rules. Nonetheless, these methods are purely computer modeling and involve the development of specific software that makes them hardly applicable to analysis within the framework of quantitative systems pharmacology. Therefore, we apply a more illustrative simplified approach of modeling of multisite phosphorylation of a protein via an approach developed earlier [18] for kinetic description of complex signaling pathways with scaffold proteins. This approach is based on the assumption of independence of phosphorylation events and combines usual kinetic modeling methods with the theory of probability of partially independent events. Here, independence means that kinetic constants for phosphorylation of a distinct site do not depend on the phosphorylation state of other sites. With the help of this approach, we may track distinct-site phosphorylation only and can compute the concentration of any microstate of tau protein from the concentrations of different sites by means of the probability theory. Such an approach is effective because we are able both to reduce combinatorial complexity and use experimental data on phosphorylation of different stand-alone sites of tau protein.

Our main goal was to develop for the first time the quantitative model of multisite (de)phosphorylation of tau protein and to elucidate the relative contribution of kinases and phosphatases to the phosphorylation of distinct sites.

## Methods

A short description of the probability-based approach for description of multisite protein phosphorylation is presented below. For a detailed description, see S1 Appendix.

Let us consider a protein with *n* phosphorylable sites. The concentration of the protein in a prefixed state (phosphorylated or unphosphorylated *i*^*th*^ site) is a macrostate *S*(*a*_*i*_) where *a*_*i*_ = 1 or 0. It is defined as a sum of microstates in which each site except *a*_*i*_ can be in a phosphorylated (*a*_*i*_ = 1) or unphosphorylated state (*a*_*i*_ = 0):

$$S(a_{i})=\sum_{a_{1}=0}^{1}\cdots \sum_{a_{i-1}=0}^{1}\sum_{a_{i+1}=0}^{1}\cdots \sum_{a_{n}=0}^{1}s(a_{1}\ldots a_{n})$$ (1)

The reduction of such a high-dimensional phosphorylation microstate space to a smaller number of functional states may be performed by a probabilistic method [18].

By definition, the probability to find the protein in a particular microstate equals to the proportion of the protein in this microstate:

$$p(a_{1}\ldots a_{n})=s(a_{1}\ldots a_{n})/S_{total}$$ (2)

If we assume independent phosphorylation of distinct sites, then microstate probability equals the product of probabilities of the corresponding macrostates:

$$p(a_{1}\ldots a_{n})=\prod_{i=1}^{n}\left(S(a_{i})/S_{total}\right)$$ (3)

Therefore, the theory of probability, when applied to multisite protein phosphorylation, links the concentration of the protein in any microstate to the concentration of distinct (un)phosphorylated sites that is measured experimentally via formulas (2) and (3). Macrostates *S*(*a*_*i*_ = 0) = *R*_*i*_ are substrates for kinases, and macrostates *S*(*a*_*i*_ = 1) = *P*_*i*_ are products of phosphorylation (see formulas below).

**Fig 2:**
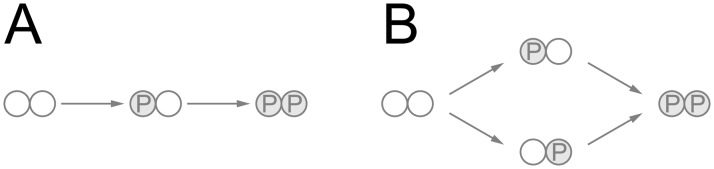
Mechanisms of multisite protein phosphorylation relevant to the independence of phosphorylation events. GSK3β phosphorylates tau via a random mechanism (B) except for sites S404 and S396, which are phosphorylated by a sequential mechanism (A). CDK5 phosphorylates all the sites via a random mechanism (B). Phosphatase PP2A dephosphorylates tau protein by a random mechanism.

This approach enabled the description of multisite phosphorylation by a random or sequential mechanism (Fig 2) avoiding combinatorial explosion of phosphorylated entities. For the random mechanism, the described approach does not require any additional assumptions. As for the sequential mechanism by which GSK3β phosphorylates S396 and S404, the concentration of S396 as a substrate for GSK3β equals the product of macrostate S396 concentration and the probability of S404 in the phosphorylated state:

$$R_{396}^{GSK3\beta }=S(a_{396}=0,a_{404}=1)=S(a_{396}=0)\cdot S(a_{404}=1)/S_{total}$$ (4)

Existing published theoretical approaches to multisite protein phosphorylation describe a situation when all phosphorylation sites are fully available for a kinase [19]. The approaches differ in the considered order of phosphorylation events. Nonetheless, as evidenced by experimental in vitro data for tau protein [20, 21], the total stoichiometry is much less than it could have been if all phosphorylable sites had been phosphorylated at least on a short experimental time scale. Below, we formulate assumptions and approximations underlying the development of the phosphorylation model for describing short-term kinetics based on experimental in vitro data:

1. Tau residues that undergo phosphorylation and are taken into account in the model were chosen based on available in vitro experimental data [20]. Phosphorylation of the following 10 residues is described in the model: T181, S199, S202, T205, T212, T217, T231, S396, S404, and S422. Given that there is no appropriate data for most of potentially phosphorylable tau sites, we incorporated a pseudoresidue into the model to describe total tau phosphorylation stoichiometry. The pseudoresidue represents all phosphorylable sites of tau protein aside from the 10 described above. Because GSK3β and CDK5 phosphorylate different tau sites, the pseudoresidues for these kinases also different too. The unknown contribution of the pseudoresidue to the total tau phosphorylation stoichiometry may be indirectly estimated as a difference between total tau phosphorylation and the contribution of measured phosphorylation of distinct sites to the stoichiometry. Pseudoresidue incorporation increases model uncertainty, and typically, data determine the advisability of its use. Equations for the pseudoresidue are analogous to equations for other sites.
2. There are “closed” and “open” states of the site for a kinase. Because experimental phosphorylation kinetic curves for some residues and total tau did not reach a plateau, we can assume that very long-term kinetics make tau residues fully available for kinases. “Closed” states of residues exist, and they are assumed to be in a very slow equilibrium with an “open” state. Hence, during a short-term phosphorylation reaction (when time is not sufficient to observe opening of a closed residue), only some tau molecules undergo phosphorylation at a specific site, depending on the proportion of initially opened sites, as confirmed by in vitro studies [21] and by incorporation of total phosphate into tau [20]. On the long-term time scale, the slow equilibrium will be shifted to the formation of the “open” state owing to phosphorylation-mediated outflow from the “open” state.
3. The reactions of phosphorylation of protein sites are independent. Residues of tau protein are phosphorylated independently from each other (except for S396 and S404, see above) but compete for binding to a kinase. Priming (phosphorylation of S214) may influence subsequent phosphorylation of other sites (positive or negative priming), potentially via the conformational change that leads to opening of the closed sites (a change of parameter α_i_ describing a residue’s extent of openness) or to an increase in kinase catalytic efficiency (*k*_*i*_).
4. Kinases that were taken into account in the model are GSK3β, PKA, CDK5, and p38γ. GSK3β and CDK5 phosphorylate all the analyzed residues of tau protein, but PKA is a priming kinase and does not phosphorylate any residues explicitly. Kinetic data for p38γ were not available, and catalytic constants for four residues (S199, T205, S396 and S404) were chosen arbitrarily so that T205 phosphorylation would be dependent strongly on p38γ activity because APP23 mice with depletion of p38γ show a lack of T205 phosphorylation [9].
5. Only protein phosphatase 2A (PP2A) is taken into account in this model. PP2A dephosphorylates all residues.

Each residue in the open state is considered a substrate, and its phosphorylation follows standard Michaelis–Menten kinetics with substrate competition (assumption 3) and K_M_ equal for all residues.

$$R_{i}^{close}\overset{slow}{↔}R_{i}^{open}\overset{fast}{\to }P_{i}$$ (5)

$$\frac{d}{dt}R_{i}^{open}=V_{i}^{close}-V_{i}^{open}-V_{i}^{phosphor}$$ (6)

$$\frac{d}{dt}P_{i}=V_{i}^{phosphor}$$ (7)

$$V_{i}^{close}=k_{i}^{close}\cdot R_{i}^{close}$$ (8)

$$V_{i}^{open}=k_{i}^{open}\cdot R_{i}^{open}$$ (9)

$$V_{i}^{phosphor}=\frac{k_{i}\cdot kinase\cdot R_{i}^{open}}{K_{M}+\sum R_{i}^{open}}$$ (10)

Partial residue phosphorylation is described by means of the initial state of the system:

$$R_{i}^{open}(0)=\alpha_{i}\cdot tau$$ (11)

$$\alpha_{i}=\{\begin{array}{ll} \alpha_{i}, & PKA=0\\ \alpha_{i}^{PKA}\text{,} & PKA=1 \end{array}$$ (12)

$$k_{i}=\{\begin{array}{ll} k_{i}, & PKA=0\\ k_{i}^{PKA}\text{,} & PKA=1 \end{array}$$ (13)

$$N=\sum \alpha_{i}$$ (14)

Here, *α*_*i*_ is a probability that residue *i* is in an open state at the initial moment of the in vitro experiment used for calibration; then *N* is stoichiometry of tau phosphorylation when the system is close to the “steady-state”. For long-term simulations, we assume tau conformational transitions leading to complete opening of the sites, i.e., all *α* parameters are equal to 1.0.

### The fitting procedure

The fitting is performed in the DBSolve Optimum software, v.36 [22]. The objective function maximum likelihood estimator was chosen under the assumption of homoscedasticity of the experimental data.

Data extraction from figures [10, 20] was performed using version 3.12 of WebPlotDigitizer (https://automeris.io/WebPlotDigitizer/index.html).

The calibration of model parameters was carried out in two main stages. At the first step, proportionality coefficient *f* (between arbitrary units and concentration) and parameters corresponding to phosphorylation (individual *k*_*i*_ and *α*_*i*_) were determined. Then, phosphorylation level for each residue was calculated as described in the experimental procedure [10] and served as initial conditions for determination of dephosphorylation parameters (individual *k*_*i*_ for phosphatase) at the second stage.

#### Fitting of phosphorylation parameters

The majority of experimental data used for construction and calibration of the model were measured for full-length tau protein. Data for model calibration included kinetics of tau phosphorylation [20] and dephosphorylation [10] at individual sites and total tau phosphate incorporation [20].

Four datasets were selected for fitting of the phosphorylation parameters: the kinetics of the phosphorylation of unphosphorylated tau or PKA-prephosphorylated tau by GSK3β or CDK5. Individual estimates of *k*_*i*_ and *α*_*i*_ were determined for each dataset; proportionality coefficient f was shared among all four datasets. Michaelis constants for GSK3β and CDK5 were fixed at 1 μM estimated from experimental data [10, 23].

There are no available experimental data to evaluate parameter *f*, but the initial value for its fitting was estimated via the following reasoning (see S1 Appendix). Because the number of sites for potential phosphorylation by GSK3β or CDK5 is greater than 10, we incorporated the pseudoresidue, which represents the sum of all other phosphorylable sites. Next, for each of the four datasets, coefficient *f* was calculated as a ratio of the sum of experimentally measured levels of phosphorylation for each of the 10 sites in arbitrary units (*E*_*i*_) to the total measured level of tau phosphorylation (*tau* ⋅ *N*), assuming that 10 sites cover all phosphorylation stoichiometry (i.e., without a pseudoresidue):

$$f=\frac{\sum E_{i}}{tau\cdot N}$$ (15)

A maximum of four calculated parameters *f* were obtained for the dataset on tau phosphorylation by CDK5 without prephosphorylation, and it was chosen as a unique initial value for subsequent simultaneous fitting to all datasets including data for total tau phosphorylation stoichiometry.

Given that some experimental curves do not show saturation of growth during short-term kinetics, parameters *α*_*i*_ were subdivided into groups according to similar values after preliminary fitting and were assumed to have a unique value inside the group in order to set the reasonable limits for parameters and behavior of the simulated curves. This approach also allowed for reducing the number of parameters and improved computational efficiency. The same reduction was applied to the number of parameters *k*_*i*_. At the last step, *k*_*i*_ and *α*_*i*_ (which remained independent) were refitted. Thus, 36 of 42 *α* parameters were placed into 11 groups based on similarity of the preliminarily fitted values (see S1 Appendix). The same procedure for catalytic constants of kinases was conducted, reducing the number of parameters from 41 to 24.

Therefore, the fitting procedure could be contingently divided into three steps: estimation of starting parameter values for fitting, preliminary fitting of all the parameters to reveal the similarity between parameters, and final fitting of the reduced set of parameters.

Analysis of the parameters’ local identifiability was carried out, and 95% confidence intervals for the fitted parameters were calculated (see S1 Appendix). All the fitted parameters were locally identifiable.

#### Fitting of dephosphorylation parameters

Initial concentrations of phosphorylated forms of residues as substrates for a phosphatase (PP2A) were calculated after calibration of phosphorylation parameters (for GSK3β and CDK5). Experimental data were available only for six residues (S199, S202, T205, T212, S396, and S404) for which catalytic constants were identified. For the remaining five residues (T181, T217, T231, S422, and the pseudo-residue) catalytic constants were equal to the averaged value of the six identified constants. The Michaelis constant was fixed at 11.6 μM taken from a literature source [10].

### Estimation of confidence intervals

These intervals were estimated for all the parameters fitted to the experimental datasets by the linearization method [24]. The estimation was performed for phosphorylation and dephosphorylation independently of each other. We carried out the following procedure (workflow):

1. Optimization of MLE (maximum likelihood estimator) in DBSolve Optimum v.36 [22].
2. Estimation of confidence intervals based on the covariance matrix using R package *dbs*.
3. For some simulations confidence bands were calculated via the Monte-Carlo approach.

### Sensitivity analysis

For selected parameters (enzyme concentrations, K_M_ and catalytic constants), 10^5^ random sets of parameters were generated from a uniform distribution. Limits for parameter generation were as follows: a) from zeros to doubled values obtained from fitting for catalytic constants related to GSK3β, CDK5, and PP2A; b) from zero to 1.0 for the level of enzymes; c) from zero to 100 for Michaelis constants and p38γ-related catalytic constants. For each set of parameters, endpoints (phosphorylation states of residues and total stoichiometry) were calculated, and then for each pair (endpoint–parameter), Pearson’s correlation coefficients were computed and presented as a heatmap.

## Results

### Description of the phosphorylation data

**Fig 3:**
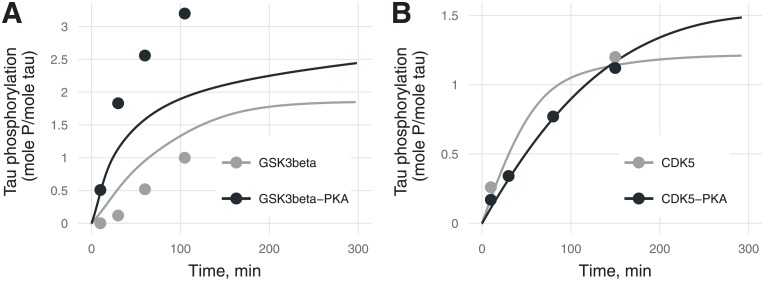
Phosphorylation stoichiometry (the ratio of phosphorylated tau to total tau, mol P/mol) for (A) GSK3β and (B) CDK5 with unphosphorylated tau (gray) or PKA-prephosphorylated tau (black) [20]. Experimental values are marked by points, and model predictions by lines. Two black and gray experimental points for CDK5 (B) coincide. Errors of experimental values were not provided by the authors [20].

**Fig 4:**
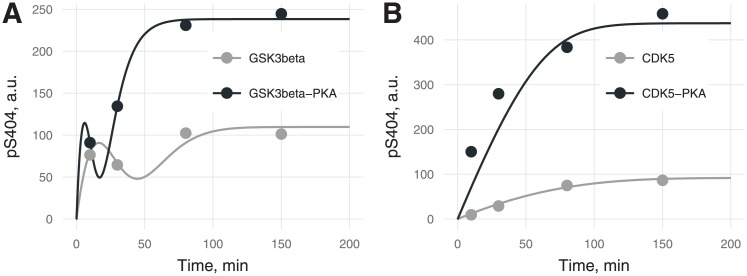
Kinetics of tau phosphorylation at S404 by (A) GSK3β or (B) CDK5 with (black) or without (gray) PKA-prephosphorylation. The transient peak in (A) is caused by sequential phosphorylation (first S404, then S396) by GSK3β in contrast to kinetics of tau phosphorylation at S404 with CDK5 when residues S396 and S404 are phosphorylated independently by a random mechanism. Errors of experimental values were not provided by the authors [20].

**Fig 5:**
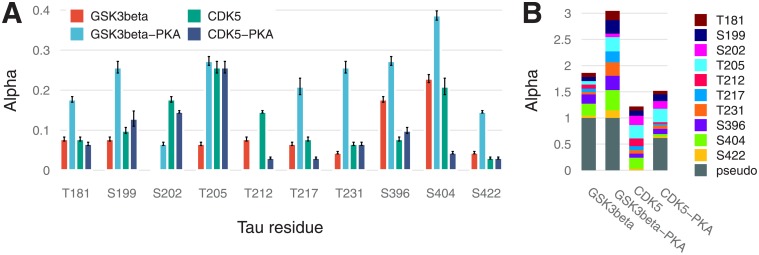
Phosphorylation kinetics of S396 (purple) and S404 (green) of tau (A) or PKA-prephosphorylated tau (B) by GSK3β. Kinetics for pS404 with 95% confidence bands are represented. Errors of experimental values were not provided by the authors [20].

Model parameters were calibrated against experimental data. Description of the data by the final model was satisfactory (see examples in Figs 3–5).

Several versions of model calibration were performed corresponding to the following hypotheses:

1. The proportion of opened residues (*α*_*i*_) depends on PKA. Data revealed that PKA-prephosphorylation influences the final level of residue phosphorylation (determined by *α*_*i*_) and the total stoichiometry that cannot be attributed to catalytic constants.
2. Kinases’ catalytic constants (*k*_*i*_) for specific residues are also PKA-dependent.

After the first stage, the data were described with insufficient quality. The second hypothesis led to over parametrization and to overestimation of total tau stoichiometry. The number of parameters was reduced by grouping as described in Methods.

Description of total tau phosphorylation stoichiometry was found to be satisfactory (Fig 3). The model predicted that PKA-mediated priming increases the stoichiometry of tau phosphorylation in the steady state by 24% for CDK5 and by 50% for GSK3β.

The majority of curves for distinct sites did not reach a steady state and inform catalytic constants better than final phosphorylation stoichiometry. Because for CDK5 there are more curves with a tendency for saturation than for GSK3β, phosphorylation by CDK5 is described better than the one by GSK3β, as expected.

**Fig 6:**
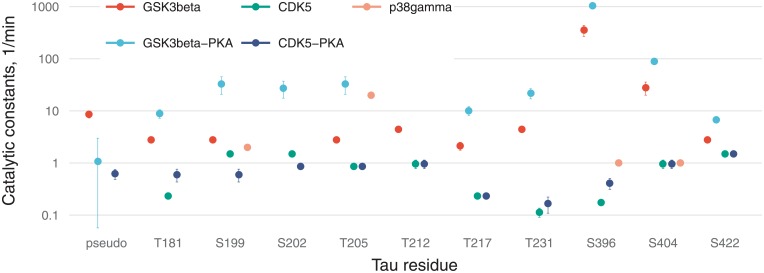
A bar chart of *α*_*i*_ parameters (proportion of opened states) for 10 sites with 95% confidence intervals (A), and a stacked bar chart for the same sites including a pseudo-residue (B).

**Fig 7:**
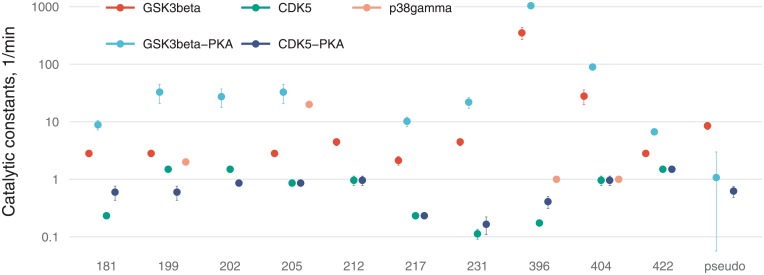
Values of catalytic constants on a logarithmic scale with 95% confidence intervals.

Fig 4 depicts differences in phosphorylation kinetics for the sequential mechanism (by GSK3β) and random mechanism (by CDK5). The random phosphorylation kinetic curve is monotonic, whereas for sequential phosphorylation, the kinetic curve shows apparent biphasic behavior (Fig 4A), which is confirmed by experimental data [21] as well as by theoretical predictions [25]. The transient peak and lower plateau (Fig 5) for site S404 in the sequential mechanism is likely to be due to the antibody detection methodology: when the second phosphorylation event inhibits the binding of detecting antibodies to the first phosphorylated site. In accordance with the above supposition, the experimentally measured level of S404 phosphorylation in the model is proportional to the difference between variable pS404 (when only S404 is phosphorylated) and variable pS396 (when S404 and S396 are phosphorylated but not detected by antibodies for pS404). This difference leads to an apparently lower plateau of S404 phosphorylation although *α*_*404*_ is higher than *α*_*396*_. Time resolution of the experiments and insufficient binding properties of site-specific antibodies do not allow us to explain such a phenomenon unambiguously. PKA-mediated priming increases the catalytic constant and *α* parameter (Figs 6 and 7), thereby causing the shift of the transient peak to the left (Fig 4A).

Phosphorylation kinetics in Fig 5 confirm S396 phosphorylation followed by that of S404. Besides, *α*_*404*_ is higher than *α*_*396*_, as expected from the sequential mechanism, although the steady state for pS396 is higher than the one for pS404. The narrow shape of the confidence bands confirms the shape of the pS404 curves, suggesting that this effect is not due to overfitting of the model.

Fig 6 illustrates residual stoichiometry (determined by *α*) for distinct sites (A) and its contributions to total tau stoichiometry (B). For six residues (S199, S202, T205, T212, T217, and T231) without PKA-prephosphorylation, the”openness” for CDK5 is higher than that for GSK3β and vice versa for the three residues (S396, S404, and S422). The pseudoresidue as a conglomeration of all other potentially phosphorylated residues is considered more”open” for GSK3β than CDK5 regardless of tau pretreatment. This situation makes tau protein a more preferable substrate for GSK3β than for CDK5, and this notion is supported by a greater number of GSK3β-phosphorylable sites [2] and catalytic constants (Fig 7). For tau phosphorylated by CDK without PKA-prephosphorylation, we assume the pseudoresidue to be absent to avoid uncertainty in the identification of parameters owing to interdependence between parameter *f* and parameters related to the pseudoresidue (*α* and *k*; see S1 Appendix). For the other three cases, the pseudoresidue increases the phosphorylation stoichiometry to the required level dictated by experimental data.

PKA-mediated priming for total tau protein phosphorylation is positive (Fig 6B), whereas for the several distinct sites (e.g., for T181, S202, T212, T217, and S404 phosphorylated by CDK5), priming is negative.

Fig 7 reveals that catalytic efficacy (in terms of *k*_*i*_) of GSK3β differs from that of CDK5 by up to three orders of magnitude (e.g., for S396). For kinase p38γ, catalytic constants were not fitted and were chosen for sensitivity analysis so that only T205 phosphorylation would be dependent strongly on p38γ activity in contrast to the other three sites.

### Description of the dephosphorylation data

**Fig 8:**
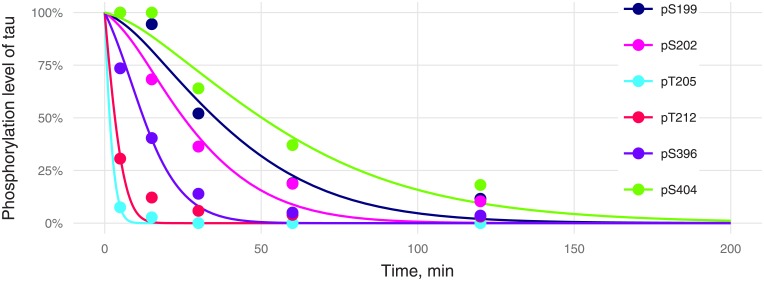
Kinetics of tau dephosphorylation at individual phosphorylation sites by PP2A. Experimental values are marked by points, and model predictions by lines. Errors of experimental values were not provided by the authors [10].

The slow dephosphorylation kinetics (S404 or S199) were described worse than fast kinetics (Fig 8). This outcome may be due to conformational changes and interdependence of dephosphorylation events. Slowly dephosphorylated residues may be hidden from phosphatases at the beginning of the dephosphorylation process, but it is unclear whether the phosphatase or detection antibody is more sensitive to tau conformation. The supposition that pS396 decreases the binding of detection antibodies to pS404 (used in the phosphorylation part of the model) could be applied here to partially explain the initial pS404 dephosphorylation delay. Indeed, let us consider two pools of tau molecules: pool 1 consisting of pS404S396 tau and pool 2 of pS404pS396 tau. Dephosphorylation of pS396 tau will transform pool 2 to pool 1 (with high affinity for detection antibodies); this situation apparently is seen as the absence of dephosphorylation of pS404 at an early stage. This effect was ignored in the model used for simulations in Fig 8.

### Simulations and sensitivity analyses

Pseudoglobal sensitivity analyses were conducted to explore 1) how the uncertainty in the output of a mathematical model can be apportioned to the different sources of uncertainty in its inputs; 2) how sensitive the different tau states are toward potential disturbances in the system. Phosphorylation states of residues and total tau phosphorylation stoichiometry were chosen as endpoints.

**Fig 9:**
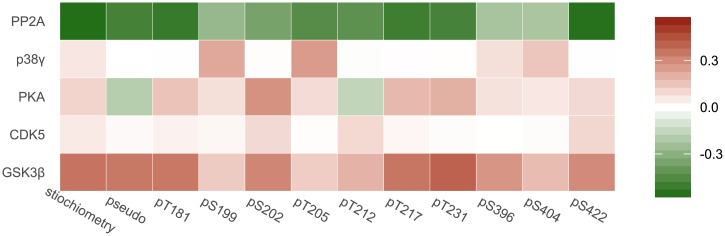
Heatmap representation of predicted sensitivity of tau residues and total tau phosphorylation (columns) to the reaction of (de)phosphorylation (row).

Our sensitivity analysis (Fig 9) revealed that the chosen endpoints and therefore distinct-site phosphorylation were sensitive to GSK3β and PP2A levels. The observed negligible cross-dependence between residues, which is assessed by Pearson’s coefficient of correlation between the level of phosphosite and catalytic constants for other sites, reflects the assumed independence of phosphorylation reactions via the random mechanism, suggesting that competition between residues makes only a minimal contribution. Of note, cross-dependence was not observed even for the pair of sites S396 and S404, which are phosphorylated sequentially by GSK3β. Nevertheless, cross-dependence between S396 and S404 was well pronounced during sensitivity analyses for early time points (data not shown). Phosphorylation of S396 is not sensitive to the catalytic constant of S404 probably because pS404 is dephosphorylated very slowly (Fig 8). Because the limits of generated parameters depend on fitted values (see Methods, Sensitivity analysis), the random sample will not contain high values of the pS404 dephosphorylation constant. Therefore, for most of the generated sets of parameters, residue S404 will have the level of phosphorylation high enough to ensure S396 phosphorylation. Total tau phosphorylation stoichiometry is most sensitive to GSK3β in comparison to other kinases because catalytic efficiency (*k*_*i*_) of GSK3β is much higher (Fig 7). We can conclude that, for instance, for a decrease in PHF1 phosphorylated tau (i.e., reduced phosphorylation on residues S396 and S404) GSK3β would be the best target, whereas pS231 may be the most sensitive biomarker for this type of therapy. PP2A activation may not lead to specific inhibition of PHF1, but may result in the decrease of pS205 concentration, which is undesirable, because this state is thought to be protective [26].

Sensitivity to the CDK5 level is unexpectedly low and could be explained by very low catalytic constants (Fig 7). During simulations of parameter sets within the chosen limits (see Methods), CDK5 does not contribute very much to the phosphorylation state in comparison with GSK3β.

## Discussion

Tau protein is an intrinsically disordered protein and has a repertoire of conformations in equilibrium. Without any modification of each residue, there is a ratio of an open state to the sum of open and closed states. Typically, this ratio is a time-dependent function and may increase or decrease during phosphorylation, thereby reflecting the variable repertoire of conformations. The ratio should approach 1.0 when phosphorylation reaches saturation (complete phosphorylation of distinct sites), which is the shared feature of published models [19], hampering application of these approaches to short-term tau protein phosphorylation. The challenging distinct feature of our approach is the partial phosphorylation of a site for describing the short-term experiments.

Implementation of partial phosphorylation of distinct tau residues enabled us to accurately reproduce the short-term kinetics of tau (de)phosphorylation reported in refs. [10, 20]. Because experimental data on phosphorylation of some residues appear linear or even exponential, it is impossible to avoid parameter redundancy when Michaelis–Menten equations are used with a product of two parameters: *α*_*i*_ and *k*_*i*_. One of the possible solutions (implemented in our model) is to reveal a similarity between the fitted values of parameters, equate them, and use shared values for such groups. Thus, grouping of similar parameters can help to overcome uncertainty when researchers use biological data with unknown or large measurement inaccuracy or in case of a lack of data. We estimated the model parameters by means of in vitro data on the phosphorylation of tau or PKA-prephosphorylated tau by GSK3β and CDK5 and determined confidence intervals for them. Overall, several groups of α-parameters have emerged (see S1 Appendix) with nonoverlapping confidence intervals (see S1 Appendix). Although they were found simply by coincidence of values, this result may have a biological meaning. Each grouping may include open-state probability for different sites and across different kinases. For example, *α*_*217*_ for CDK is equal to *α*_*181*_, *α*_*199*_, and *α*_*212*_ for GSK3β. This coincidence can reveal that some specific tau conformation exists for which these residues are opened simultaneously. That is why their *α* parameters are equal and reflect the probability of such a conformation.

It is well known that hyperphosphorylation of tau is associated with tau aggregation [27], but not all phosphorylation events are associated with toxicity [28]. Increased phosphorylation of tau occurs during fetal development and transiently in hibernating mammals. One phosphorylation pattern leads to destabilization of microtubules [29] and tau aggregation, but other patterns may be protective against degeneration or apoptosis [26]. Phosphorylation’s influence on aggregation or toxicity is often revealed via site-specific pseudophosphorylation.

Our pseudoglobal sensitivity analysis can be considered an analysis of sensitivity of specific tau states to potential therapeutic interventions (kinase inhibition or phosphatase activation).

Phosphorylation of tau at S396 is significantly reduced by a GSK3β knockdown, whereas phosphorylation of T181 not reduced by suppression of GSK3β in SH-SY5Y cells [30]. In many clinical studies, the level of phosphorylated residue T181 is measured. The ARGO study showed that GSK3β inhibitor tideglusib decreases phosphorylation of T181 and S396 but offers no clinical benefit [31]. Although pT181 is a good biomarker to discriminate between AD and non-AD pathologies [32, 33], our approach may help to find new biomarkers with increased diagnostic power to more specifically distinguish between different tauopathies with different phosphosite fingerprints. It has been discovered that during the phosphorylation of tau by PKA, the first residue appearing to be phosphorylated is pS214, followed by pS208 and pS324 and finally pS409 and pS416 [34]. Although PKA phosphorylates several tau sites, only S214 is found to be phosphorylated to a saturation level under the experimental conditions for preparation of PKA-prephosphorylated tau [35]. Consequently, we can conclude that phosphorylation of S214, followed by conformational changes, causes PKA-dependent priming. Only residue pT231 is necessary for the binding of the AT180 antibody, with a dissociation constant of 30 nM, whereas phosphorylation of S235 does not interfere with this binding [36]. Phosphorylation of T205 by p38γ was found to be required for disruption of PSD-95–tau–Fyn complexes, thus preventing excitotoxicity and amyloid β toxicity [26].

Various kinds of cross-talk between kinases and phosphatases subtly regulating tau phosphorylation have been investigated [37, 38]. PP2A activates GSK3β by dephosphorylating pS9-GSK3β [8] in a PP2A-Leu309 methylation–independent manner [39], and this influence was incorporated into our model for simulations and sensitivity analyses. GSK3β stimulates the inhibitory phosphorylation of PP2A at Y307 (pY307-PP2A) [40]. This phenomenon was not implemented into the model because this effect is indirect and difficult to evaluate. Although GSK3β is primed not only by PKA but also by CDK5, and probably vice versa, such reciprocal priming is not implemented into the model because of the lack of quantitative data. Sequential phosphorylation of tau at S404 and S396 can be considered the priming of GSK3β by itself.

Experimental data on the dynamics of key phosphorylation events are essential for the development of accurate quantitative and mechanistic models of multisite phosphorylation and priming. On the other hand, experimental data often are not very amenable to calibration of model parameters and require making some assumptions. Model predictions may help to improve experimental design. For example, it would be better to increase the time resolution of phosphorylation kinetics, especially at the beginning of the reaction, and to continue the reaction until a plateau is reach. This strategy would have confirmed the transient peak during S404 phosphorylation by GSK3β (Fig 4A) and allowed us to validate the grouping of parameters *α*_*i*_ and *k*_*i*_. Besides, it is impossible to evaluate the contribution of GSK3β autophosphorylation to kinetic constants of tau phosphorylation; therefore, determination of the GSK3β phosphorylation state during the reaction should provide some helpful information. Biochemical approaches, such as immunoblotting with phosphospecific antibodies, are routinely used for monitoring of (previously identified) phosphorylation sites. Mathematical description requires quantitative information that can hardly be obtained by immunoblotting. There are some limitations of the antibody-based approach for monitoring of phosphorylation events. First, there is the issue of availability of specific antibodies for distinct sites that can be potentially phosphorylated. Second, resolution is low due to spatial closeness of phosphorylation sites. Typically, antibody epitopes are 4–12 amino acid residues long [41], suggesting that an epitope may overlap with the GSK3β consensus motif (S/T)XXX(pS/pT), for example. Therefore, the binding of antibodies to distinct sites can be affected by the milieu dependent on conformations and phosphorylation of neighboring sites. For example, PHF1 antibodies bind to phosphorylated residues S396 and S404. For GSK3β in the (S/T)XXX(pS/pT) consensus motif, the sites are close to each other, creating obstacles when priming is studied. Limitations of antibodies may be partially overcome by means of miniantibodies, which are less sensitive to tau conformation and to the target epitope environment, or by other methodsMass spectrometric data for protein phosphorylation may be useful for kinetic analysis and modeling although rather few applications exist at present. Time-resolved high-resolution nuclear magnetic resonance (NMR) spectroscopy has been employed recently to study mechanistic characteristics of multisite protein phosphorylation [19, 35].

## Conclusions

In the present study, a mathematical mechanism-based model of multisite (de)phosphorylation of tau protein was developed. The main features of the model are partial phosphorylation of tau residues, and merging random and sequential mechanisms of multisite phosphorylation within the framework of the probability-based approach assuming independent phosphorylation events. This approach illustrates how analysis of in vitro kinetics allows for the choice of better candidates and for a greater understanding of relations between therapeutic targets and biomarkers. The model describes phosphorylation of tau or PKA-prephosphorylated tau by GSK3β and CDK5 and dephosphorylation by PP2A, thereby accurately reproducing the data on short-term kinetics of tau (de)phosphorylation. Our results suggest that kinase inhibition may prevent tau hyperphosphorylation more specifically, e.g., on PHF sites, which are the key biomarkers of AD pathology. The model may serve as a submodel for subsequent development of a systems pharmacological model of tau pathology for research into biologically relevant challenges.

## Supporting information

S1 Appendix (DOCX)

S1 File The model in SBML format. (XML)

S1 Table Dataset for the model calibration. (XLSX)
