# Correction: A mathematical model of multisite phosphorylation of tau protein

**DOI:** 10.1371/journal.pone.0194002

**Published:** 2018-03-01

**Authors:** Alexander Stepanov, Tatiana Karelina, Nikolai Markevich, Oleg Demin, Timothy Nicholas

Fig 5, Fig 6, and Fig 7 are incorrect. Please see the correct figures here.

**Fig 5:**
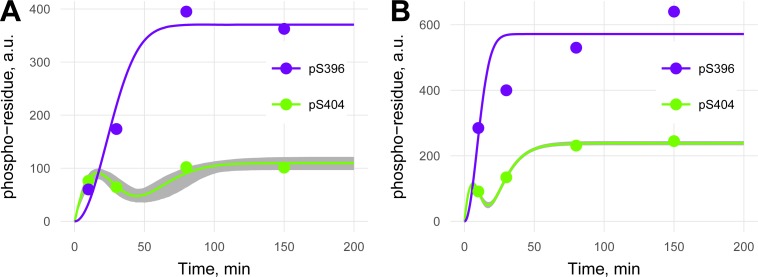
**Phosphorylation kinetics of S396 (purple) and S404 (green) of tau (A) or PKA-prephosphorylated tau (B) by GSK3β.** Kinetics for pS404 with 95% confidence bands are represented. Errors of experimental values were not provided by the authors [20].

**Fig 6:**
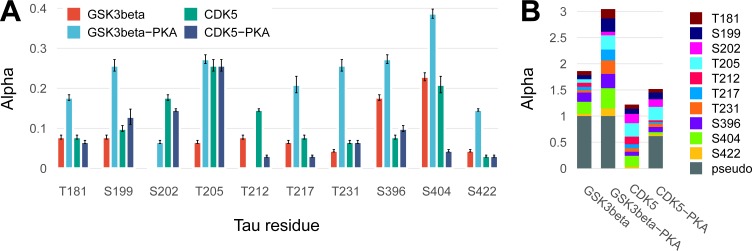
**A bar chart of αi parameters (proportion of opened states) for 10 sites with 95% confidence intervals (A), and a stacked bar chart for the same sites including a pseudo-residue (B)**.

**Fig 7:**
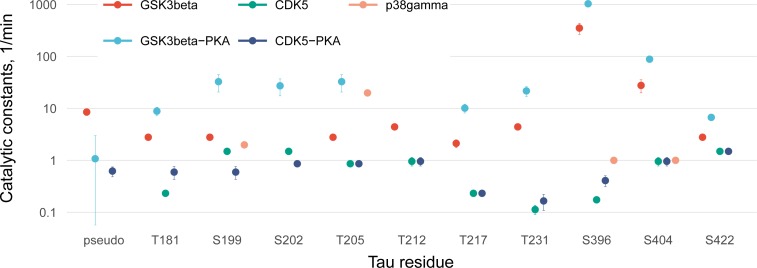
Values of catalytic constants on a logarithmic scale with 95% confidence intervals.
