# Supplementary material for: A mathematical model of multisite phosphorylation of tau protein
Source: PLoS One. 2018 Feb 6;13(2):e0192519. doi: 10.1371/journal.pone.0192519 (PMC5800643; doi:10.1371/journal.pone.0192519)
Supplement: S1 Appendix — (DOCX) [file pone.0192519.s001.docx]

**Supplementary**

A mathematical model of multisite phosphorylation of tau protein

Alexander Stepanov*, Tatiana Karelina*, Nikolai Markevich*^&^, Oleg Demin*, Timothy Nicholas^#^

*InSysBio, Nauchny proezd 19, 117246 Moscow, Russia

^#^Pfizer Global R&D, Groton, CT 06340, USA

^&^current affiliation: Institute of Theoretical and Experimental Biophysics of RAS, 142290 Pushchino, Russia

Tatiana Karelina ORCID ID: 0000-0003-4643-3023

Oleg Demin ORCID ID: 0000-0003-3606-050X

Corresponding Author:

Alexander Stepanov

E-mail: [stepanov@insysbio.ru](mailto:stepanov@insysbio.ru)

Tel.: +79067603962

ORCID ID: 0000-0002-9113-9440

Table of contents

[Random phosphorylation mechanism description 2](#_Toc493849918)

[Phosphorylation of two independent sites 2](#_Toc493849919)

[Microstates description 2](#_Toc493849920)

[Macrostates description 3](#_Toc493849921)

[Relationship between micro- and macrovariables 4](#_Toc493849922)

[Phosphorylation of *n* independent sites 5](#_Toc493849923)

[Microstates description 5](#_Toc493849924)

[Macrostates description 6](#_Toc493849925)

[Relationship between micro- and macrovariables 6](#_Toc493849926)

[Sequential phosphorylation mechanism description 6](#_Toc493849927)

[Fitting results 6](#_Toc493849928)

[Model ODE system 9](#_Toc493849929)

[List of variables 10](#_Toc493849930)

[Tables of parameters 11](#_Toc493849931)

[Limitations 13](#_Toc493849932)

[Estimation of parameter *f* 13](#_Toc493849933)

[References 15](#_Toc493849934)

# **Random phosphorylation mechanism description**

Here the approach of dimensionality reduction of high-dimensional microstate spaces to a smaller number of functional states based on the theory of probability and the assumption of independence of distinct sites phosphorylation is described.

Consider a protein with *n* phosphorylable sites. Each site can be in one of two states denoted by *a_i_* = 0 (unphosphorylated site) or *a_i_* = 1 (phosphorylated site). Such protein has 2^n^ possible microstates, which can be designated as *s(a_1_…a_n_)*. The concentration of the protein in each state at any time may be expressed by the time-dependent function *s(a_1_…a_n_, t)*. In order to simplify computation of this function describing numerous microstates of the protein, we introduce the macrovariables *S(a_i_, t)* that represent the sum of microstates:

$S(a_{i})=\sum_{a_{1}=0}^{1} \cdots\sum_{a_{i-1}=0}^{1} \sum_{a_{i+1}=0}^{1} \cdots\sum_{a_{n}=0}^{1} s(a_{1}...a_{n})$ (S1)

Each of these macrovariables describes states of only one *i*-site and can be consider as the concentration of this site in a particular state (phosphorylated or not). This means that the protein concentration *S(a_i_)* in a particular macrostate (*a_i_*) is the sum of all forms of a protein.

The next step in this approach is a transition from the concentrations to the probabilities. Indeed, the fractional concentration of protein in the microstate *s(a_1_…a_n_, t)* corresponds to the probability to find the protein in this state:

$p(a_{1}...a_{n},t)=s(a_{1}...a_{n},t)/S_{tot}$ (S2)

where the total protein concentration (*S_tot_*) is expressed as follows:

$S_{tot}=\sum_{a_{1}=0}^{1} \cdots\sum_{a_{n}=0}^{1} s(a_{1}...a_{n})=\sum_{a_{i}=0}^{1} S(a_{i})$ (S3)

It has been shown [1] that for independent phosphorylation of distinct sites, finding the protein in a certain microstate *s(a_1_…a_n_)* equals the product of the probabilities of the corresponding states for each site:

$s(a_{1}...a_{n},t)/S_{tot}=\prod_{i=1}^{n} (S(a_{i},t)/S_{tot})$ (S4)

We assume the rate of distinct site (de)phosphorylation could be described by enzyme kinetics and approximated by Michaelis-Menten equation.

## Phosphorylation of two independent sites

### **Microstates description**

Kinetic scheme representing microdescription of the system is shown in Fig. S1A, where *s*(00), *s*(10), *s*(01) and *s*(11) are the microstates of the protein with two sites.

The following differential equations describe changes in the concentration of microstates presented at the kinetic scheme:

(S5)

Assuming both sites are independent, (de)phosphorylation of one site does not dependent on the state (phosphorylated or not) of another one. This means that kinetic parameters of 1^st^ and 4^th^ as well as 2^nd^ and 3^rd^ steps at the kinetic scheme in Fig. S1A are equal. Therefore, the rates of (de)phosphorylation for both sites can be expressed as follows:

$\begin{matrix} v_{1}^{k}=\frac{k_{1}^{k}\cdot E^{k}\cdot s(00)}{K_{M_{1}}^{k}+s(00)+s(01)}\text{ }v_{1}^{p}=\frac{k_{1}^{p}\cdot E^{p}\cdot s(10)}{K_{M_{1}}^{p}+s(10)+s(11)} \\ v_{2}^{k}=\frac{k_{2}^{k}\cdot E^{k}\cdot s(10)}{K_{M_{2}}^{k}+s(00)+s(10)}\text{ }v_{2}^{p}=\frac{k_{2}^{p}\cdot E^{p}\cdot s(11)}{K_{M_{2}}^{p}+s(01)+s(11)} \\ v_{3}^{k}=\frac{k_{2}^{k}\cdot E^{k}\cdot s(00)}{K_{M_{2}}^{k}+s(00)+s(10)}\text{ }v_{3}^{p}=\frac{k_{2}^{p}\cdot E^{p}\cdot s(01)}{K_{M_{2}}^{p}+s(01)+s(11)} \\ v_{4}^{k}=\frac{k_{1}^{k}\cdot E^{k}\cdot s(01)}{K_{M_{1}}^{k}+s(00)+s(01)}\text{ }v_{4}^{p}=\frac{k_{1}^{p}\cdot E^{p}\cdot s(11)}{K_{M_{1}}^{p}+s(10)+s(11)} \end{matrix}$ (S6)


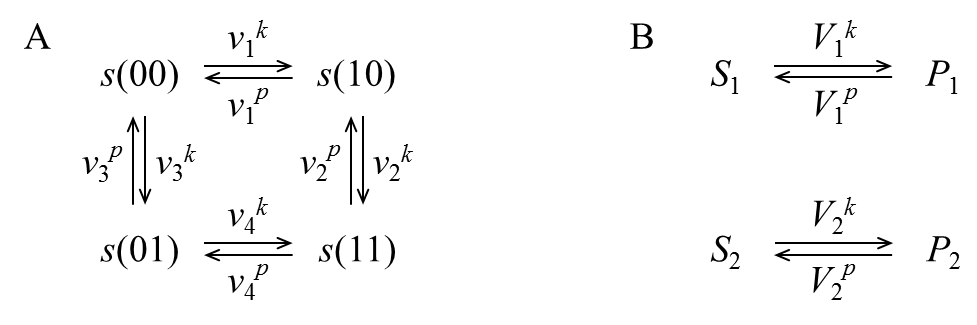


*Fig. S1. Kinetic scheme of (de)phosphorylation of a protein with two sites in terms of micro- (A) and macrostates (B).*

### **Macrostates description**

Summing up differential equations (S5) for microstates results in emergence of macrostates and macrovariables:

(S7)

Designate the sums of microvariables as macrovariables:

$\begin{matrix} S_{1}=s(00)+s(01) \\ S_{2}=s(00)+s(10) \\ P_{1}=s(10)+s(11) \\ P_{2}=s(01)+s(11) \end{matrix}$ (S8)

Then, differential equations in macro-variables are expressed as follows:

(S9)

where

$\begin{matrix} V_{1}^{k}=v_{1}^{k}+v_{4}^{k}=\frac{k_{1}^{k}\cdot E^{k}\cdot S_{1}}{K_{M_{1}}^{k}+S_{1}} \\ V_{2}^{k}=v_{2}^{k}+v_{3}^{k}=\frac{k_{2}^{k}\cdot E^{k}\cdot S_{2}}{K_{M_{2}}^{k}+S_{2}} \\ V_{1}^{p}=v_{1}^{p}+v_{4}^{p}=\frac{k_{1}^{p}\cdot E^{p}\cdot P_{1}}{K_{M_{2}}^{p}+P_{1}} \\ V_{2}^{p}=v_{2}^{p}+v_{3}^{p}=\frac{k_{1}^{p}\cdot E^{p}\cdot P_{2}}{K_{M_{1}}^{p}+P_{2}} \end{matrix}$ (S10)

In this case the kinetic scheme presented in Fig. S1A can be dissected into two independent kinetic schemes that describe separately (de)phosphorylation of 1^st^ and 2^nd^ sites with the rates $V_{1}^{k},V_{2}^{k},V_{1}^{p},V_{2}^{p}$as shown on Fig. S1B.

### **Relationship between micro- and macrovariables**

Fractional concentrations of protein (normalized by total protein concentration) in any micro- or macrostate equal probabilities of finding of the protein in these micro- and macrostates. Therefore, the theory of probability for description of multisite phosphorylation can be used. It has been shown earlier [1] that probabilities of microstates:

$\begin{matrix} s(00)=S(00)/S_{tot} \\ s(01)=S(01)/S_{tot} \\ s(10)=S(10)/S_{tot} \\ s(11)=S(11)/S_{tot} \end{matrix}$ (S11)

can be expressed for independent sites as the product of probabilities of macrostates:

$\begin{matrix} s(00)=\frac{S_{1}}{S_{tot}}\cdot\frac{S_{2}}{S_{tot}} \\ s(01)=\frac{S_{1}}{S_{tot}}\cdot\frac{P_{2}}{S_{tot}} \\ s(10)=\frac{P_{1}}{S_{tot}}\cdot\frac{S_{2}}{S_{tot}} \\ s(11)=\frac{P_{1}}{S_{tot}}\cdot\frac{P_{2}}{S_{tot}} \end{matrix}$ (S12)

Therefore, the concentration of the protein in any microstate can be expressed through the concentration of distinct phosphorylated and unphosphorylated sites measured experimentally:

$\begin{matrix} S(00)=S_{1}\cdot S_{2}/S_{tot} \\ S(01)=S_{1}\cdot P_{2}/S_{tot} \\ S(10)=P_{1}\cdot S_{2}/S_{tot} \\ S(11)=P_{1}\cdot P_{2}/S_{tot} \end{matrix}$ (S13)

By induction, this method, which combines theories of kinetics and probability, can be applied for description of independent (de)phosphorylation of any *N*-site protein.

## Phosphorylation of *n* independent sites

In this section we present general formulas describing (de)phosphorylation of a protein with *n* sites. It is assumed that all the sites are (de)phosphorylated independently from each other.

### **Microstates description**

*s*(*a_1_…a_n_*) – the function that describes microstates of the *n*-site protein, where *a_i_* = 0 indicates *i*-th unphosphorylated site and *a_i_* = 1 denotes *i*-th phosphorylated site. The protein has 2^n^ different microstates.

Let us consider processes involved in (de)phosphorylation of *j^th^*-residue of the protein:

$S(a_{1}\ldots a_{j}=0\ldots a_{n})\leftrightarrow_{V_{i}^{p}}^{V_{i}^{k}} S(a_{1}\ldots a_{j}=1\ldots a_{n})$ (S14)

ODE system describing dynamics of the two states is presented below:

(S15)

(De)phosphorylation of *i^th^*-site with the rates $V_{i}^{k}$ and $V_{i}^{p}$ is a change in one of *a_i_* (*i*≠*j*).

### **Macrostates description**

In this case, macrovariables are the following:

$\begin{matrix} S_{i}=\sum_{a_{1}=0}^{1} \ldots\sum_{a_{i-1}=0}^{1} \sum_{a_{i+1}=0}^{1} \ldots\sum_{a_{n}=0}^{1} S(a_{1}\ldots a_{i}=0\ldots a_{n}) \\ P_{i}=\sum_{a_{1}=0}^{1} \ldots\sum_{a_{i-1}=0}^{1} \sum_{a_{i+1}=0}^{1} \ldots\sum_{a_{n}=0}^{1} S(a_{1}\ldots a_{i}=1\ldots a_{n}) \\ V^{K}=\sum_{i=1}^{n} V_{i}^{k} \\ V^{P}=\sum_{i=1}^{n} V_{i}^{p} \end{matrix}$ (S16)

The total kinetic scheme in macrovariables presents also separate phosphorylation-dephosphorylation cycles for distinct sites.

### **Relationship between micro- and macrovariables**

It has been shown earlier [1] that for independent phosphorylation of different sites finding the protein in a certain microstate *s*(*a_1_…a_n_*) equals the product of the probabilities of the corresponding states for each site (equation S3).

# **Sequential phosphorylation mechanism description**

Tau residues S396 and S404 are phosphorylated by GSK3β sequentially. Site S396 become as substrate for the kinase when S404 is already phosphorylated. From equations (S13) we could adopt one related to this case:

$S(01)=S_{1}\cdot P_{2}/S_{tot}$ (S17)

where S_1_ is a concentration of residue S396 in ‘open’ state, P_2_ is a concentration of phosphorylated S404 residue and S_tot_ is a total tau concentration.

# **Fitting results**


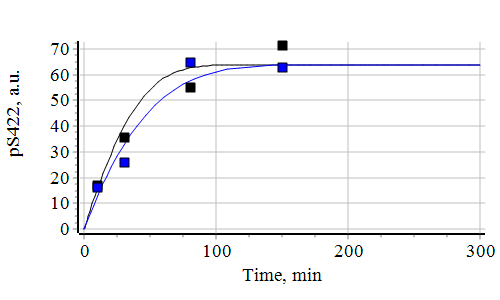

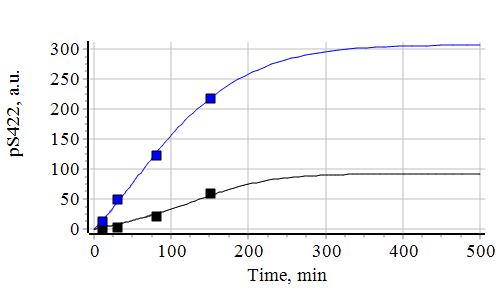


Figure S2. Phosphorylation kinetics of the residue S422 of tau (black) or PKA-prephosphorylated tau (blue) by CDK5 (left) or GSK3β (right).


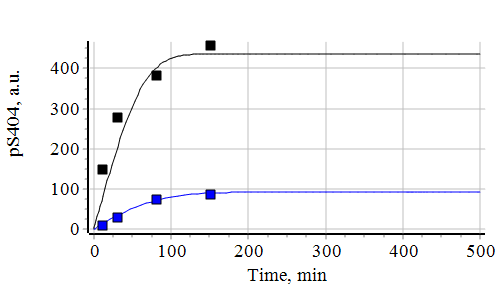

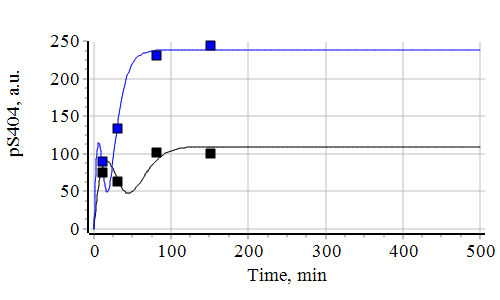


Figure S3. Phosphorylation kinetics of the residue S404 of tau (black) or PKA-prephosphorylated tau (blue) by CDK5 (left) or GSK3β (right).


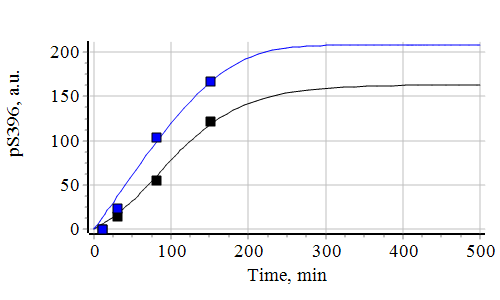

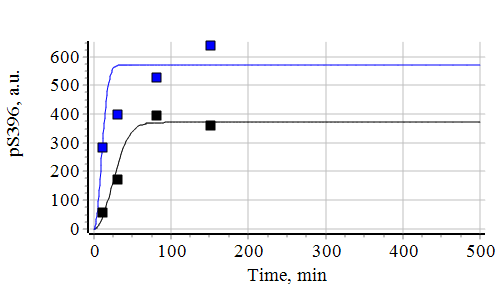


Figure S4. Phosphorylation kinetics of the residue S396 of tau (black) or PKA-prephosphorylated tau (blue) by CDK5 (left) or GSK3β (right).


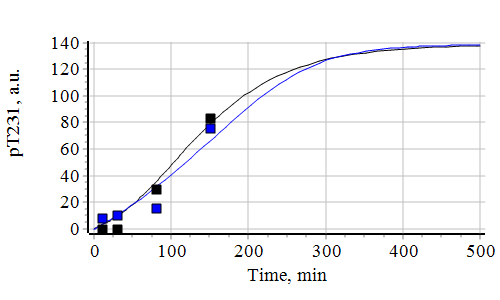

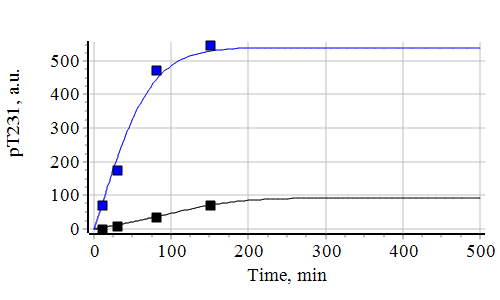


Figure S5. Phosphorylation kinetics of the residue T231 of tau (black) or PKA-prephosphorylated tau (blue) by CDK5 (left) or GSK3β (right).


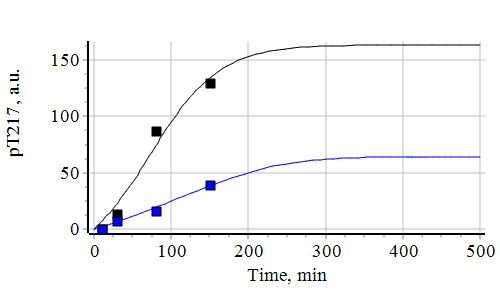

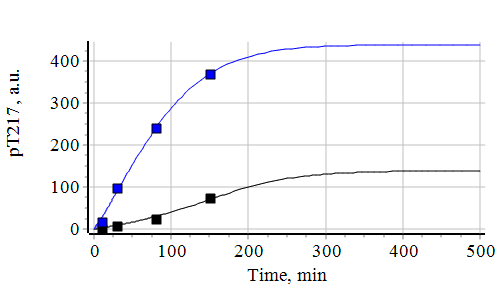


Figure S6. Phosphorylation kinetics of the residue T217 of tau (black) or PKA-prephosphorylated tau (blue) by CDK5 (left) or GSK3β (right).


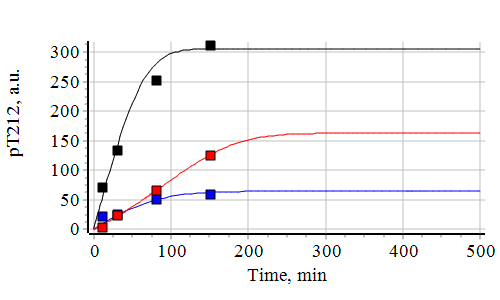


Figure S7. Phosphorylation kinetics of the residue T212 of tau (black and red) or PKA-prephosphorylated tau (blue) by CDK5 (black and blue) or GSK3β (red). PKA-prephosphorylation abolishes residue phosphorylation by GSK3β.


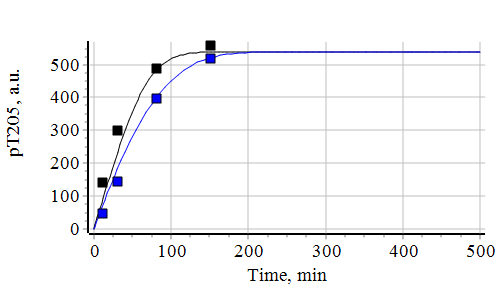

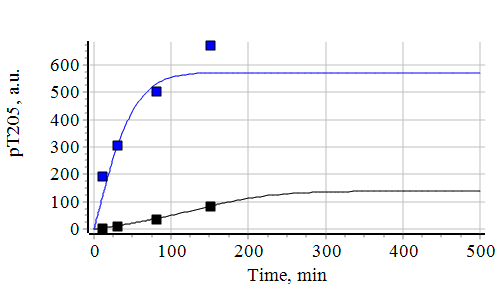


Figure S8. Phosphorylation kinetics of the residue T205 of tau (black) or PKA-prephosphorylated tau (blue) by CDK5 (left) or GSK3β (right).


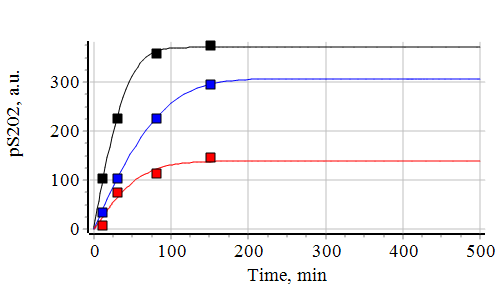


Figure S9. Phosphorylation kinetics of the residue S202 of tau (black) or PKA-prephosphorylated tau (blue and red) by CDK5 (black and blue) or GSK3β (red). GSK3β phosphorylates S202 without PKA-prephosphorylation at negligible level.


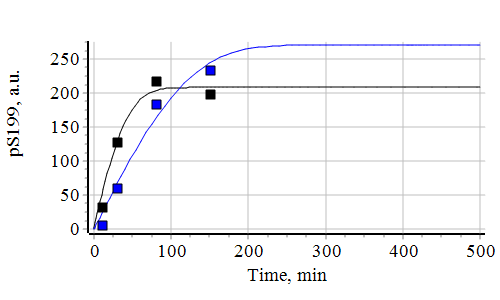

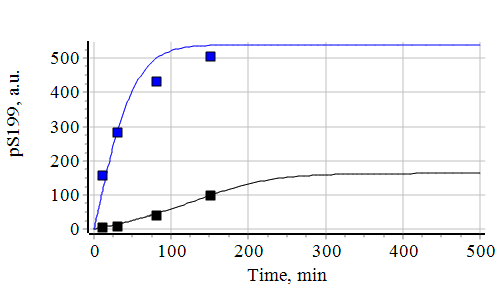


Figure S10. Phosphorylation kinetics of the residue S199 of tau (black) or PKA-prephosphorylated tau (blue) by CDK5 (left) or GSK3β (right).


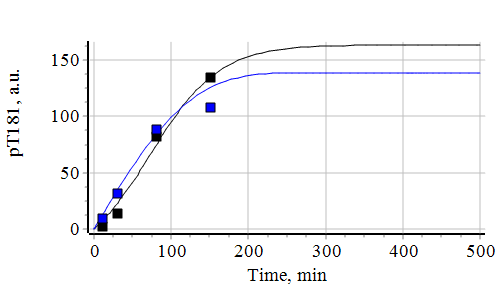

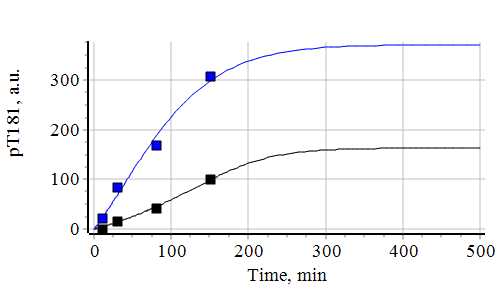


Figure S11. Phosphorylation kinetics of the residue T181 of tau (black) or PKA-prephosphorylated tau (blue) by CDK5 (left) or GSK3β (right).

# **Model ODE system**

In general, equations for calibration of (de)phosphorylation model are following:

$$v_{i}^{k}=\frac{k_{i}^{k}\cdot E^{k}\cdot\alpha_{i}\cdot tau}{K_{M}^{k}+tau\cdot\sum\alpha_{i}}$$

$$\alpha_{i}=\left\{\begin{matrix} \alpha_{i},\text{ }PKA=0 \\ \alpha_{i}^{PKA}\text{, }PKA=1 \end{matrix}\right.$$

$$k_{i}^{k}=\left\{\begin{matrix} k_{i}^{k},\text{ }PKA=0 \\ k_{i}^{PKA}\text{, }PKA=1 \end{matrix}\right.$$

$$v_{i}^{p}=\frac{k_{i}^{p}\cdot E^{p}\cdot p_{i}}{K_{M}^{p}+\sum p_{i}}$$

Here PKA denotes the presence of PKA preincubation before beginning of the experiment, and *α* denotes parameters for portions of opened states for each site (see explanation in the main text). Example for T181 residue is given below:

$$v_{181}^{gsk}=\frac{k_{181}^{gsk}\cdot GSK3\beta\cdot\alpha_{181}^{gsk}\cdot tau}{K_{M}+tau\cdot\sum\alpha_{i}^{gsk}}$$

$$v_{181}^{cdk}=\frac{k_{181}^{cdk}\cdot CDK5\cdot\alpha_{181}^{cdk}\cdot tau}{K_{M}+tau\cdot\sum\alpha_{i}^{cdk}}$$

$$v_{181}^{pp2a}=\frac{k_{181}^{pp2a}\cdot PP2A\cdot p_{181}}{K_{M}^{pp2a}+\sum p_{i}}$$

Analogous equations describe other ten residues including pseudo-residue.

The model equations used for long-term predictions are slightly differing from above that are used for fitting. For this case parameters alpha equaled to one as well as kinase p38γ was incorporated. In general, equations are following:

$$v_{i}^{k}=\frac{\left(k_{i}^{k}\cdot(1-PKA)+k_{i}^{pka,k}\cdot PKA\right)\cdot E^{k}\cdot r_{i}}{K_{M}^{k}+\sum r_{i}}$$

$$v_{i}^{p}=\frac{k_{i}^{p}\cdot E^{p}\cdot p_{i}}{K_{M}^{p}+\sum p_{i}}$$

An example for T181 residue is following:

$$v_{181}^{gsk}=\frac{\left(k_{181}^{gsk}\cdot(1-PKA)+k_{181}^{pka,gsk}\cdot PKA\right)\cdot GSK3\beta\cdot r_{181}}{K_{M}+\sum r_{i}}$$

$$v_{181}^{cdk}=\frac{\left(k_{181}^{cdk}\cdot(1-PKA)+k_{181}^{pka,cdk}\cdot PKA\right)\cdot CDK5\cdot r_{181}}{K_{M}+\sum r_{i}}$$

$$v_{181}^{p38}=\frac{k_{181}^{p38}\cdot p38\gamma\cdot r_{181}}{K_{M}^{p38}+\sum r_{i}}$$

$$v_{181}^{pp2a}=\frac{k_{181}^{pp2a}\cdot PP2A\cdot p_{181}}{K_{M}^{pp2a}+\sum p_{i}}$$

Analogous equations describe other ten residues including pseudo-residue.

# **List of variables**

$r_{181},r_{199},r_{202},r_{205},r_{212},r_{217},r_{231},r_{396},r_{404},r_{422},r_{000}$ - unphosphorylated residues in ‘open’ state.

$p_{181},p_{199},p_{202},p_{205},p_{212},p_{217},p_{231},p_{396},p_{404},p_{422},p_{000}$ - phosphorylated residues.

# **Tables of parameters**


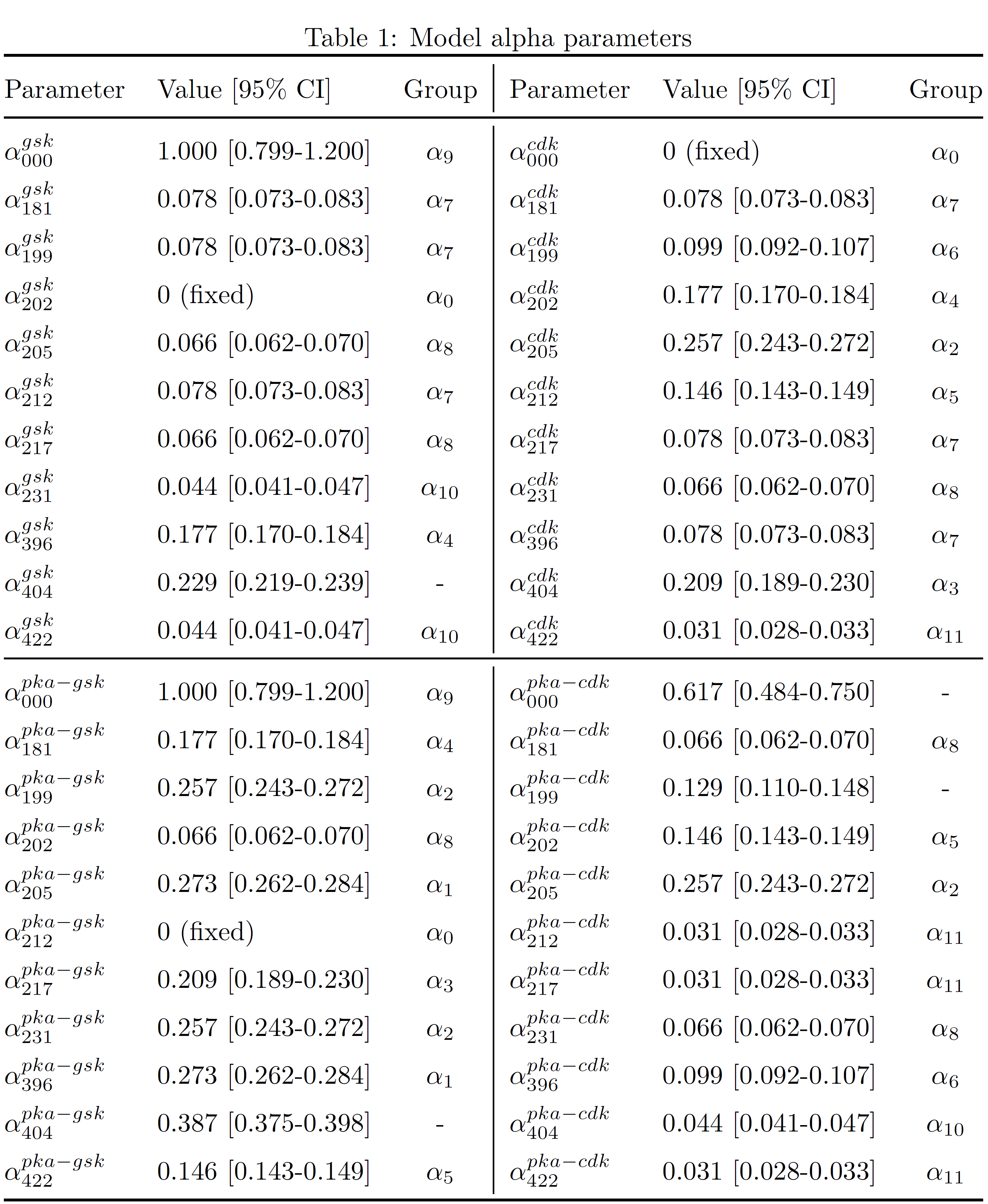


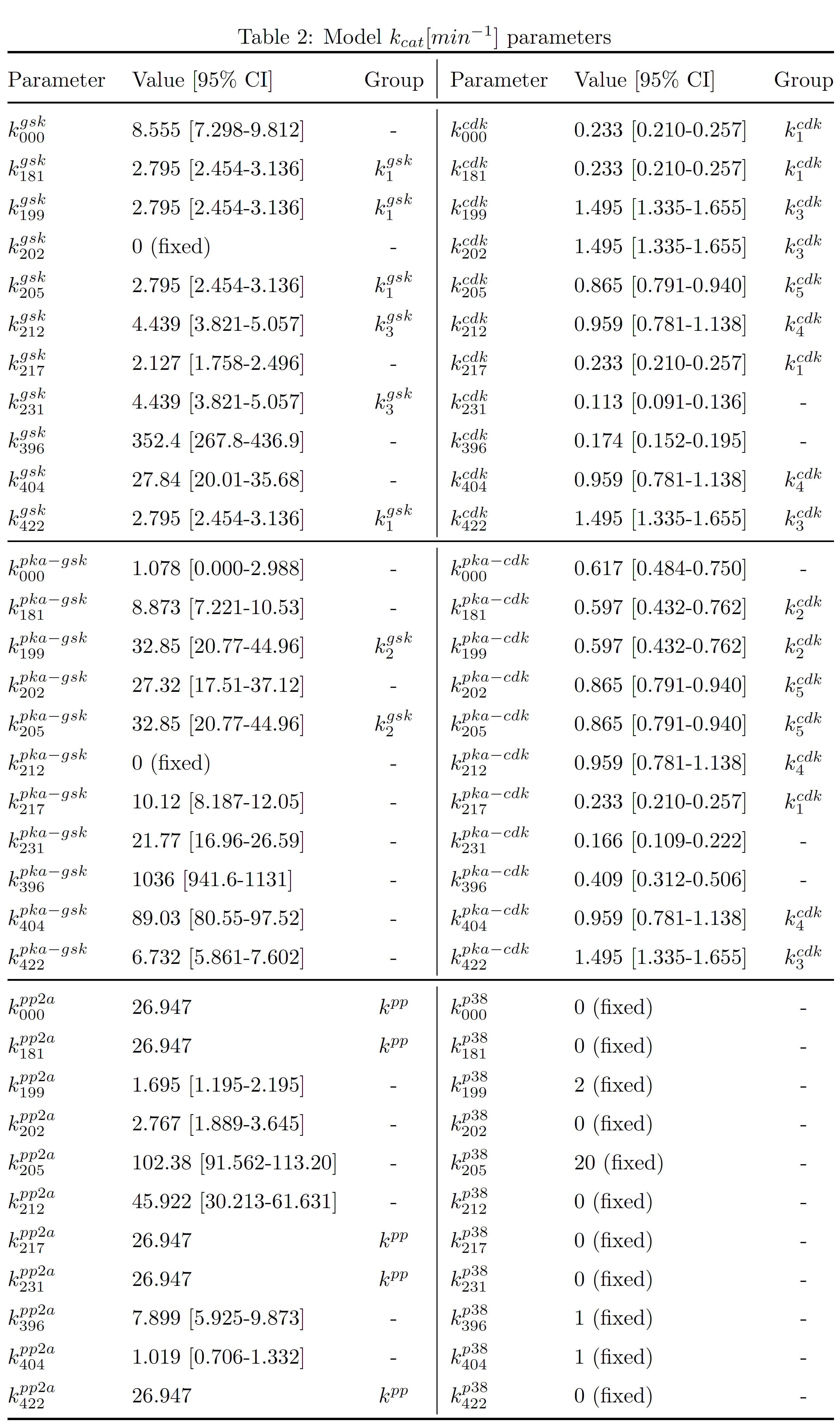

$$k^{pp}=(k_{199}^{pp2a}+k_{202}^{pp2a}+k_{205}^{pp2a}+k_{212}^{pp2a}+k_{396}^{pp2a}+k_{404}^{pp2a})/6$$

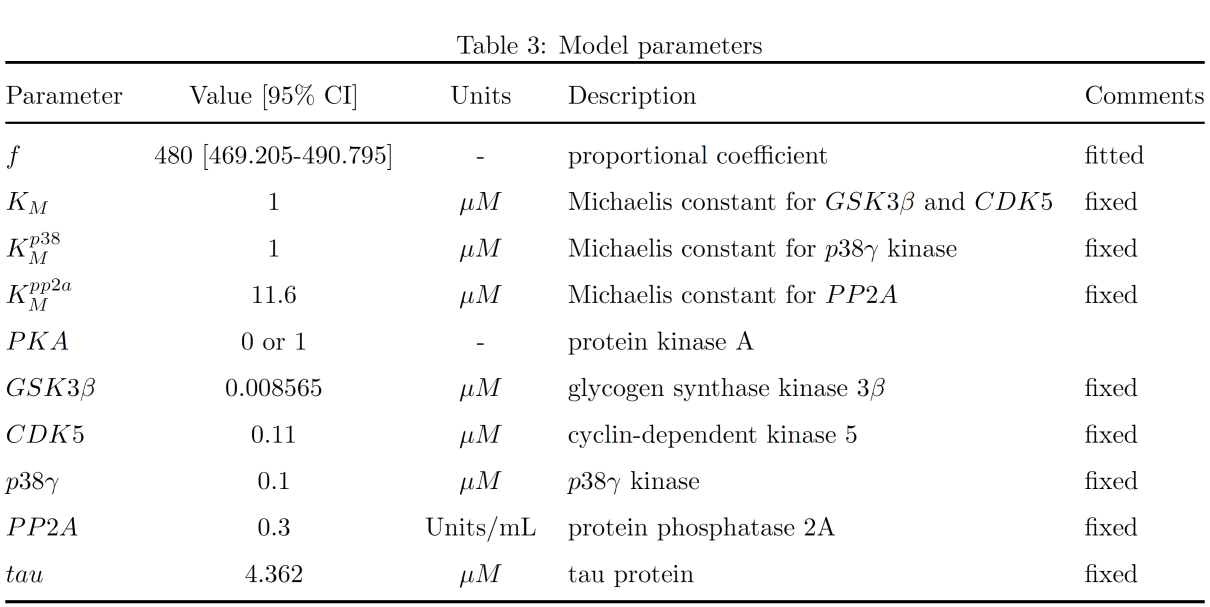


# **Limitations**

Partial residues phosphorylation implemented into the model is a simplification in order to describe short-term kinetics replacing parameters for long-term conformational transitions. A limitation of such simplification is arisen when we describe more than one kinase (but not phosphatases) in the system simultaneously.

Consider a residue phosphorylation by two kinases. Initial concentration of a residue in “open” state (substrate) for the first kinase equals $\alpha^{k1}\cdot tau$ and for the second one – $\alpha^{k2}\cdot tau$:

$\left\{\begin{matrix} r^{k1}(t)=\alpha^{k1}\cdot tau-p(t) \\ r^{k2}(t)=\alpha^{k2}\cdot tau-p(t) \end{matrix}\right.$ (S17)

At the end of the second reaction $p=\alpha^{k2}\cdot tau$. Then

$r^{k1}=\alpha^{k1}\cdot tau-\alpha^{k2}\cdot tau=(\alpha^{k1}-\alpha^{k2})\cdot tau$ (S18)

At specified conditions (here $\alpha^{k1}\leq\alpha^{k2}$) substrate concentration for one of kinases will be negative. So this approach has application limitations. Phosphatases in the system may keep the substrates at the “non-negative” level.

# **Estimation of parameter *f***

For fitting phosphorylation part of the model there are four datasets corresponding to phosphorylation of unphosphorylated or PKA-prephosphorylated tau by GSK3β or CDK5. For each dataset, there is a set of *kcat_i_* and *α_i_* and proportion coefficient *f* between arbitrary units and concentration is shared for all four datasets. Before fitting parameters, we have estimated proportion coefficients from following reasoning. Although the number of phosphorylable sites much more than 10 we have assumed that these 10 sites cover the most part of phosphorylation stoichiometry. It means that we have to choose maximal *f* (*f_max_*) among ones (*f*) calculated for each of four datasets. For dataset with *f_max_* we assumed that 10 residues determine total stoichiometry of tau phosphorylation (there are no other phosphorylable sites). Then for remaining three datasets we added a pseudoresidue (r_000_) that is represent all other phosphorylable sites in one. This pseudosite helps to increase the stoichiometry to required level dictated by experimental data.

By definition, proportion coefficient is the ratio of experimentally measured level of phospho-site in arbitrary units to concentration of phosphor-site in μM:

$f=\frac{\sum E_{i}}{\sum p_{i}}$ (S19)

Where *E_i_* is experimentally measured level of phospho-site in a.u. and *p_i_* is concentration of phospho-site. Ideally, *f* should be calculated near steady-state. But in available data the most curves do not reach steady-state and we took last experimental points to estimate *f*.

$\sum p_{i}=tau\cdot\sum\alpha_{i}=tau\cdot N$ (S20)

Where N – experimentally measured total tau phosphorylation stoichiometry. Than,

$f=\frac{\sum E_{i}}{tau\cdot N}$ (S21)

Consider dataset of tau phosphorylation by CDK5. On Fig. 2 [2] stoichiometry curves for unphosphorylated and PKA-prephosphorylated tau are coincided that is N_1_=N_2_. Sum of *E_i_* for unphosphorylated tau (without prephosphorylation) is more than for PKA-prephosphorylated tau therefore we choose maximal *f* and added pseudo-site r_000_ in the case of PKA-prephosphorylated tau in order to fit the sum of phosphorylation level of distinct sites to total tau stoichiometry.

$\sum E_{i}\approx f\cdot tau\cdot N=const$ (S22)

$\sum E_{i}^{1}=\sum\left(E_{i}^{2}+r_{000}\right)$ (S23)

Because experimental measurements have unknown errors (experimental points have no errors) and to confirm our calculations, parameters *f* were fitted against four datasets independently. Maximal fitted *f* parameter was obtained for CDK5 without prephosphorylation and differs from estimated values approximately 2%.

Maximal *f* corresponds to CDK5. Indeed, it seems reasonable because GSK3β phosphorylates more residues than CDK5 *in vitro* [3].

# **References**

[1] N. M. Borisov, N. I. Markevich, J. B. Hoek, and B. N. Kholodenko, “Signaling through receptors and scaffolds: independent interactions reduce combinatorial complexity.,” *Biophys. J.*, vol. 89, no. 2, pp. 951–66, Aug. 2005.

[2] F. Liu *et al.*, “PKA modulates GSK-3beta- and cdk5-catalyzed phosphorylation of tau in site- and kinase-specific manners.,” *FEBS Lett.*, vol. 580, no. 26, pp. 6269–74, Nov. 2006.

[3] D. P. Hanger, B. H. Anderton, and W. Noble, “Tau phosphorylation: the therapeutic challenge for neurodegenerative disease.,” *Trends Mol. Med.*, vol. 15, no. 3, pp. 112–9, Mar. 2009.
